# GDF-15 and immune resistance: A new frontier in non-small cell lung cancer immunotherapy

**DOI:** 10.1016/j.pccm.2026.07.001

**Published:** 2026-09-04

**Authors:** Jun Lu, Hua Zhong, Baohui Han

**Affiliations:** a Department of Respiratory and Critical Care Medicine, Shanghai Chest Hospital, Shanghai Jiao Tong University School of Medicine, Shanghai 200030, China; b Shanghai Key Laboratory of Thoracic Tumor Biotherapy, Shanghai Chest Hospital, Shanghai Jiao Tong University School of Medicine, Shanghai 200030, China; c Shanghai Institute of Thoracic Oncology, Shanghai Chest Hospital, Shanghai Jiao Tong University School of Medicine, Shanghai 200030, China; d Translational Medical Research Platform for Thoracic Oncology, Shanghai Chest Hospital, Shanghai Jiao Tong University School of Medicine, Shanghai 200030, China; e Department of Bio-bank, Shanghai Chest Hospital, Shanghai Jiao Tong University School of Medicine, Shanghai 200030, China

**Keywords:** GDF-15, Immunotherapy resistance, NSCLC, TME remodeling, Immunotherapy rechallenge

Dear Editor,

Immunotherapy has revolutionized first-line cancer treatment, but a significant hurdle remains: 50% to 85% of patients experience immune resistance.^1^ This phenomenon reflects a tumor’s ability to evade the immune system, severely compromising therapeutic outcomes.^2^ Consequently, overcoming this resistance and enhancing the efficacy of immunotherapies represent two of the most critical challenges in oncology today. Non-small cell lung cancer (NSCLC) accounts for approximately 85% of all lung cancer cases, and in this setting, the challenge of immune resistance is particularly acute. Although multiple immune checkpoint inhibitors (ICIs) have been approved as standard-of-care therapies, a substantial proportion of patients either fail to respond initially or eventually acquire resistance. This underscores an urgent unmet need for novel therapeutic strategies in this prevalent malignancy.

Growth differentiation factor 15 (GDF-15), originally identified in the late 1990s as macrophage inhibitory cytokine-1 (MIC-1), was first investigated for its roles in cardiovascular disease, pregnancy tolerance, and metabolic regulation, before garnering significant attention in oncology and immunology in the 2010s. Highly overexpressed in both placental tissues and solid tumors, GDF-15 plays a pivotal role in immune modulation.^2^ In the tumor microenvironment (TME), it facilitates immune evasion by fostering an immunosuppressive milieu—a function analogous to its physiological role in promoting fetal–maternal tolerance to placental alloantigens.^1^ This immunosuppressive function is co-opted by tumors in pathological contexts, most notably in cancers such as NSCLC, where GDF-15 overexpression strongly correlates with adverse clinical outcomes, including poor prognosis and accelerated disease progression. These observations establish GDF-15 as a critical mediator of immune resistance and a compelling therapeutic target in oncology.

Mechanistic studies have established GDF-15 as a pleiotropic modulator of the TME, driving immune evasion through its effects on multiple immune cell populations.^2^ Elevated GDF-15 disrupts the maturation and function of dendritic cells (DCs) by suppressing nuclear factor κB (NF-κB) signaling, which in turn downregulates major histocompatibility complex (MHC) class II and co-stimulatory molecules essential for antigen presentation.^3^ This reduction in antigen-presenting capacity impairs the activation of cluster of differentiation 4-positive (CD4⁺) and cluster of differentiation 8-positive (CD8⁺) T cells, thereby compromising tumor immunogenicity. This process is thought to involve a transforming growth factor-β receptor type II (TGF-βRII)/activin receptor-like kinase 5 (ALK-5)-dependent but glial cell line-derived neurotrophic factor family receptor alpha-like (GFRAL)-independent pathway, although this mechanism has yet to be definitively confirmed in lung cancer. Beyond impairing their activation, GDF-15 also directly induces an exhausted phenotype in T cells by upregulating immune checkpoints like programmed cell death protein 1 (PD-1) and cytotoxic T-lymphocyte–associated protein 4 (CTLA-4), which suppresses their proliferation and cytotoxicity. Furthermore, it promotes the expansion and activation of regulatory T (T_reg_) cells, which further dampen anti-tumor immunity through the secretion of immunosuppressive cytokines such as TGF-β and interleukin-10 (IL-10).^2^

GDF-15 also modulates key innate immune players within the TME.^4^ It polarizes macrophages toward a tumor-promoting M2 phenotype through janus kinase/signal transducer and activator of transcription 6 (JAK/STAT6)- and peroxisome proliferator-activated receptor gamma (PPARγ)-driven metabolic reprogramming. Concurrently, it suppresses natural killer (NK) cell cytotoxicity by inhibiting activating receptor signaling and granzyme B release, although the specific receptor mediating this effect on NK cells remains undefined.

Adding another layer of complexity, GDF-15 is implicated in the metabolic reprogramming of the TME.^5^ By activating the activating transcription factor 4 (ATF4) pathway in response to amino acid deprivation, it links metabolic stress to immune suppression. Importantly, a functional dichotomy exists: while GDF-15’s systemic metabolic effects are mediated by the canonical GFRAL–rearranged during transfection (RET) axis in the hindbrain, its local immunomodulatory functions in the TME appear to operate through putative peripheral receptors. Preclinical studies suggest potential mechanisms such as integrin inhibition (e.g., lymphocyte function-associated antigen 1 [LFA-1]) or cross-talk with erythroblastic leukemia viral oncogene homolog (ErbB)/cluster of differentiation 44 (CD44)/cluster of differentiation 48 (CD48). These diverse changes collectively remodel the TME into an immunosuppressive state, further suppressing the activation and function of cytotoxic T cells and NK cells. Notably, all these GDF-15-mediated immunosuppressive mechanisms—affecting both adaptive and innate immunity as well as metabolic homeostasis—are robustly observed in NSCLC, establishing this cancer as a prime example of GDF-15-driven immune resistance.

Given the prominent role of GDF-15 in mediating immune resistance, this cytokine has emerged as a promising therapeutic target in oncology. In a *Nature* paper, Melero et al^1^ demonstrated that neutralizing GDF-15 can overcome resistance to anti-PD-1 and anti-programmed death-ligand 1 (PD-L1) therapy in solid tumors including NSCLC, establishing a rational combination strategy. The authors reported findings from Phase I and IIa clinical trials evaluating dual targeting of GDF-15 and PD-1 in patients with immune-resistant solid tumors who had experienced disease progression following prior PD-(L)1-based therapy.^1^

In the phase I clinical trial, patients with advanced metastatic solid tumors received visugromab, a GDF-15-neutralizing antibody, in combination with nivolumab, an anti-PD-1 antibody. A standard “3 + 3” dose-escalation design confirmed an acceptable safety profile for the combination. Among the 25 enrolled patients, 12 experienced grade 3 treatment-emergent adverse events (TEAEs). Clinically, 3 patients achieved a partial response (PR) and 3 exhibited stable disease (SD). Mechanistically, blockade of GDF-15 drives extensive T cell infiltration into the tumor tissue. The infiltrating T cell populations include CD4^+^, forkhead box P3-negative (CD4⁺ FOXP3⁻) T cells (observed in 7 of 14 patients) and CD8^+^ T cells (8 of 15 patients). This was also accompanied by an expansion of proliferating cluster of differentiation 3-positive, Ki-67-positive (CD3⁺ Ki-67⁺) T cells (8 of 15 patients) and cytotoxic CD3^+^, granzyme B-positive (CD3⁺ GZMB⁺) T cells (5 of 10 patients).

Building on these findings, the team extended their investigation into a phase IIa clinical trial, enrolling 27 patients with NSCLC and 27 patients with urothelial carcinoma (UC). For NSCLC patients, the median treatment duration was 3.9 months. Anti-tumor activity included 2 complete responses (CR) and 2 PRs, representing an exploratory efficacy signal in this small cohort. However, given the limited sample size, these responses should be interpreted cautiously and considered hypothesis-generating rather than definitive evidence of clinical benefit. Grade ≥3 TEAEs occurred in 16 patients (∼60%), underscoring the need for careful risk–benefit assessment. The most common systemic grade ≥3 TEAEs included infections (14.8%), musculoskeletal disorders (14.8%), and respiratory events (14.8%).^1^ For UC patients, the median treatment duration was 5.4 months, with an objective response rate (ORR) of 18.5%. Grade ≥3 TEAEs were reported in 14 patients (51.9%).^1^ Patients with elevated baseline GDF-15 levels exhibited reduced proportions of PD-L1^+^ tumor cells, CD8^+^ T cells, and proliferating cluster of differentiation 45-positive, Ki-67^+^ (CD45⁺ Ki-67⁺) immune cells.^1^ Notably, immune cell composition varied significantly between molecular subtypes of bladder cancer (BLCA) based on GDF-15 expression. In the basal squamous subtype, a stronger negative correlation between GDF-15 levels and immune infiltration was observed, further underscoring the immunosuppressive effects of GDF-15 on the TME. Across all evaluable participants in phase I and IIa trials, these findings consistently supported the role of GDF-15 in inhibiting T cell extravasation into the TME. Collectively, phase I/IIa data validate that visugromab plus nivolumab restores tumor T-cell infiltration and yields preliminary anti-tumor activity in PD-1/PD-L1 blockade-refractory NSCLC and UC. However, the limited sample size and notable toxicity profile highlight the need for larger stratified trials to identify which patient subsets derive the greatest benefit from this GDF-15-targeting strategy.

Beyond the visugromab (CTL-002) trials discussed, several other GDF-15-targeting agents are under clinical investigation, underscoring the growing interest in this pathway. These GDF-15 neutralizing antibodies include ponsegromab (NCT05546476 and NCT06989437), NGM120 (NCT04068896 and NCT07033026), NGM438 (NCT05311618), AV380 (NCT05865535) and GFS202A (NCT06898255) (Table 1).

**Table 1:** Overview of clinical trials evaluating GDF-15-targeting therapies in solid tumors, cancer-associated cachexia, and healthy subjects.

Therapeutic agent	Target	Phase	State	Condition	Combination	NCT Number
Visugromab	GDF-15	IIb	Recruiting	Advanced NSCLC	Anti-PD-1 (pembrolizumab) plus chemotherapy	NCT07098988
Visugromab	GDF-15	II	Active, not recruiting	Muscle-invasive bladder cancer	Anti-PD-1 (nivolumab)	NCT06059547
Visugromab	GDF-15	II/III	Not yet recruiting	Cancer-associated cachexia	–	NCT07112196
Ponsegromab	GDF-15	I	Completed	Healthy adults	–	NCT05685264
Ponsegromab	GDF-15	II	Completed	Cancer, cachexia, and elevated GDF-15	–	NCT05546476
Ponsegromab	GDF-15	IIb/III	Not yet recruiting	Cachexia and metastatic pancreatic ductal adenocarcinoma	–	NCT06989437
NGM120	GDF-15	I	Completed	Healthy adults	–	NCT03392116
NGM120	GDF-15	I/II	Completed	Solid tumors	–	NCT04068896
NGM120	GDF-15	II	Recruiting	Colorectal cancer	–	NCT07033026
NGM438	GDF-15	I/Ib	Active, not recruiting	Advanced solid tumors	Anti-PD-1 (pembrolizumab)	NCT05311618
AV-380	GDF-15	Ib	Recruiting	Cancer patients with cachexia	–	NCT05865535
GFS202A	GDF-15/IL6	I	Recruiting	Advanced solid tumors	–	NCT06898255

GDF-15, Growth differentiation factor 15; IL-6, Interleukin 6; NCT, National clinical trial; NSCLC, Non-small cell lung cancer; PD-1, Programmed cell death protein 1; –, Not applicable.

Collectively, targeting GDF-15 represents a promising strategy to overcome immune resistance in cancer therapy, especially in NSCLC, where unmet clinical needs persist. By counteracting its immunosuppressive functions, it may be possible to restore effective anti-tumor immune responses. Ongoing clinical trials and a deeper mechanistic understanding of GDF-15 are expected to drive the development of targeted therapies with the potential to transform NSCLC treatment. Continued research and innovation will be essential to addressing key challenges in cancers such as NSCLC, ultimately improving outcomes for patients.

## Funding

This work was supported by grants from the National Natural Science Foundation of China (Nos. 82473102 & 82272913 & 82301980); the foundation of Shanghai Key Laboratory of Thoracic Tumor Biotherapy (No. 2025SZ1001); and the foundation of Chinese Society of Clinical Oncology (No. Y-2019AZZD-0355 & Y-QL2019-0125).

## CRediT authorship contribution statement

**Jun Lu:** Writing – review & editing, Writing – original draft, Funding acquisition, Conceptualization. **Hua Zhong:** Supervision, Funding acquisition, Conceptualization. **Baohui Han:** Supervision, Funding acquisition, Conceptualization.

## Declaration of competing interest

The authors declared that they have no known competing financial interests or personal relationships that could have appeared to influence the work reported in this paper.
